# Bone Scan Is of Doubtful Value as a First Staging Test in the Primary Presentation of Prostate Cancer

**DOI:** 10.5402/2012/585017

**Published:** 2012-11-05

**Authors:** Lina M. Carmona Echeverria, Lawrence Drudge-Coates, C. Jason Wilkins, Gordon H. Muir

**Affiliations:** ^1^Department of Urology, King's College Hospital, 2nd floor Hambleden Wing, Denmark Hill, London SE19 2BY, UK; ^2^Radiology Department, King's College Hospital, London, UK

## Abstract

*Purpose*. To determine whether axial MR imaging could replace bone scan as the primary staging test in newly diagnosed CaP. *Material and Methods*. We reviewed retrospectively all bone scans (*n* = 1201) performed in newly diagnosed CaP patients from 2000 to 2010 in a single tertiary academic center. We recorded patient age, ethnicity, PSA at diagnosis, TNM stage, Gleason score, alkaline phosphatase, bone scan results and axial imaging if available. *Results*. Mean patient age was 72 years (41–96), mean PSA and alkaline phosphatase were 268.9 ng/mL and 166 IU/L, respectively. Patients were divided in four groups according to possible bony metastases on bone scan. Group 1: Negative, no metastases demonstrated. Group 2: Positive, metastases only in pelvis and/or lumbar spine. Group 3: Positive, widespread metastases including pelvis and lumbar spine. Group 4: Positive, distant metastases without pelvic or lumbar spine abnormalities. Group 4 patients were analyzed in detail, two had possible disease that was detected only outside the pelvic and lumbar spine, unfortunately follow up images were insufficient to confirm the nature of the lesions. *Conclusions*. Although bone scan is a useful investigation to confirm and monitor metastasic CaP, our data suggests that axial MR imaging is an adequate primary staging study in untreated disease. Bone scan is unnecessary if CT or MRI of the pelvis and abdomen are clear of metastases.

## 1. Introduction

Prostate cancer was the most common cancer in the UK in 2008, accounting for 24% of all new male cancer diagnosis [1], and is responsible for around 12% of the deaths annually due to cancer (10.168 in 2008). It is calculated that 85% of the patients who die of prostate metastatic cancer have axial skeleton involvement [2], and the presence of these determines the treatment, prognosis, and possibly outcome. It is therefore a priority to determine which patients have developed bone compromise. It is also important to identify patients with little or no risk of metastases to avoid unnecessary investigations and procedures [3–5]. 

Currently, bone scan is the most commonly used test to diagnose bone metastases [5–7], although it has a low specificity due to the lack of precision in differentiating benign disease from metastatic cancer [2]. There are several studies demonstrating that only certain patients require a bone scan [3–5, 7–11] once the diagnosis has been reached (PSA level above 20 ng/mL, Gleason 4, T3-T4 disease). Also numerous studies have shown that both MRI [12–14] and CT scan [13] have a higher sensitivity and specificity than bone scan in the diagnosis of metastases (including bone metastases as well as lymph node and visceral involvement). 

Despite all the evidence that other imaging techniques might contribute more to early and accurate diagnosis of bone metastases, bone scan remains the initial staging test in most practices [7, 15]. The costs secondary to unnecessary diagnostic tests could be reduced if one test could accurately exclude metastases. The aim of this study was to determine the likelihood of presenting with metastatic bone deposits in an area that is not scanned with abdominopelvic imaging techniques, in order to assess the possibility of replacing bone scan as first line in patients with newly diagnosed CaP.

## 2. Materials and Methods

A retrospective analysis of all bone scans (*n* = 1201) performed on newly presenting patients with prostate cancer seen at King's College Hospital, London between 2000 and 2010 was carried out. We recorded patient's age, ethnicity, PSA on diagnosis, TNM stage, Gleason score, and serum alkaline phosphatase at diagnosis. 83 patients were excluded due either to the bone scans having been repeated in the same patient or patients being erroneously classified in the records as being new to our hospital, leaving 1201 newly presenting patients for the period.

**Figure 1:**
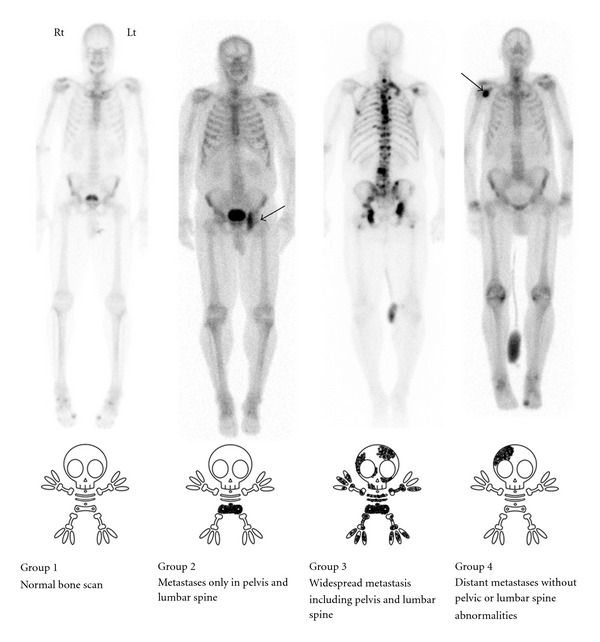
Schematic bone scan classification system.

These patients were divided into four groups according to the report of the initial bone scan (Figure 1) as follows:

 Group 1: negative, no metastases demonstrated.
 Group 2: positive, metastases only in the bony pelvis and/or lumbar spine.
 Group 3: positive, patients with widespread metastases including the area specified in group 2.
 Group 4: positive, but no metastases in pelvis or lumbar spine.

The bone scans were classified as a “high diagnostic certainty” reading, when the radiology clearly stated a diagnosis of metastatic disease, or “possible bony metastases” when the report suggested further evaluation by comparison with other imaging techniques due to diagnostic uncertainty. All bone scans which were not regarded as definitive were reviewed by a consultant radiologist (JW), a urology consultant (GM), and a urology specialist trainee (LC).

TNM staging from any axial imaging or histopathology was recorded for comparison with bone scan results.

The data was stored in an Excel Spreadsheet and analyzed with SPSS.

## 3. Results

The mean age was 72 years on diagnosis (41–96). 57.8% (*n* = 695) of the patients were White and 38.3% (*n* = 460) were Black, with 3.7% (*n* = 45) of Asian background.

**Figure 2:**
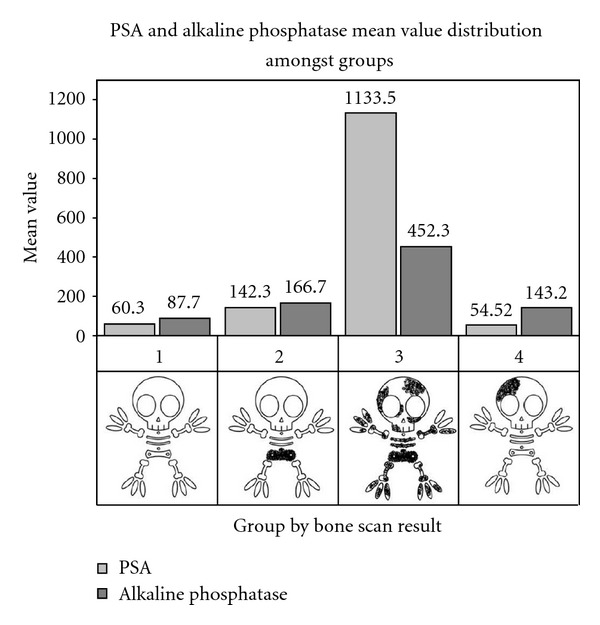
Representation of the distribution of the mean PSA and alkaline phosphatase in the different groups.

Mean PSA was 268.9 ng/mL (0.5–106931) with a median of 21 ng/mL (Figure 2). Mean alkaline phosphatase was 166.0 IU/L (7–2755) with a median of 84 IU/L (Figure 2). Note that the PSA values in Ggroup 4 are lower compared to the other Groups, including Group 1, also the alkaline phosphatase values are only just above the normal range (30–130 IU/L).

Using the UICC staging criteria (2002) 22.5% of the patients were classified as T1 (*n* = 271), 35.0% as T2 (*n* = 421), and 32.7% as T3 (*n* = 393). 73.8% of the patients did not have a lymph node status recorded (*n* = 887), 18.3% were N0 (*n* = 220) and 7.8% were classified as N+ (*n* = 94). Regarding M stage, nearly three quarters (73.6% *n* = 884) had no evidence of metastasis, 22.3% (*n* = 268) were found to have bone metastasis and 3.8% (*n* = 46) were diagnosed with visceral and/or bone metastases. 

22.0% of patients had a Gleason sum 6 (*n* = 265), 39.3% Gleason sum 7 (*n* = 473), and 28.9% Gleason sum >7 (*n* = 348). A small number of patients either had no locally verified Gleason sum or had been diagnosed on clinical grounds without biopsy (*n* = 36, 2.99%.)

Regarding the proposed classification system by bone scan result (Table 1), 820 (68.2%) of patients had no metastasis on bone scan and 11.3% (*n* = 136) had metastases only in the lumbar spine or pelvic area. 18.5% of patients had widespread metastasis (*n* = 223). Only 22 patients (1.8%) had a possible bone metastasis without pelvic or lumbar spine abnormalities on bone scan. 

**Figure 3:**
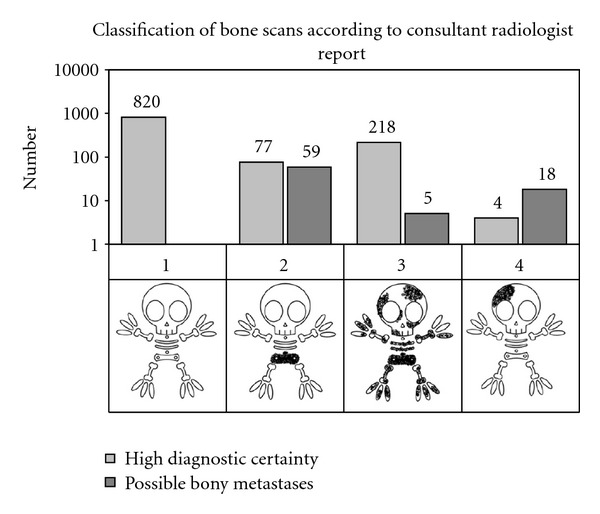
Classification of the bone scans according to consultant radiologist report.

Regarding bone scan classification by diagnostic certainty, 93.1% had “high diagnostic certainty” and 8% were classified as “possible bony metastases” (Figure 3), with further imaging advised to confirm findings. 

As expected negative bone scans (Group 1) had a high confidence. With possible deposits only outside the lumbar spine and pelvis (Group 4), 3/4 of scans were reported with a low diagnostic utility correlating with the high sensitivity and low specificity of bone scan. With positive findings at unusual sites alone (e.g., skull), false positives are more likely, and correlative imaging is commonly suggested.

Regarding further imaging modalities performed, we found that in Group 1, a total of 427 patients were studied with other imaging studies (accounting for 539 studies in total), within the next year. 8 of those patients were found to have metastatic disease or lymph node compromise where the bone scan was been negative. CT scan detected metastatic deposits in bone, lymph nodes, and visceral organs in four patients, while 4 patients were diagnosed with bulky lymphadenopathy or bone compromise on MRI scan of the pelvis and abdomen. Since the majority of these studies were done for local staging only in low-risk patients, a true false negative rate cannot be gained from this data.

As seen in Figure 3, there were 59 patients in Group 2 where the radiologist report was “possible bony metastases.” We reviewed all of these patients and found that 30 of them (50%) had degenerative changes mainly in lumbar spine, 18.6% (*n* = 11) were diagnosed with Paget's disease and 10 (16.9%) were negative on all studies. Only 2 were positive, one with liver metastasis and one with bone metastasis (3.3%). Regarding the bone scans classified as “high diagnostic certainty,” there was only one false positive, confirmed as degenerative disease by spinal MRI. In Group 3 with multiple metastases including lumbar spine and pelvis, no false positive diagnoses were made. 

All the images of the patients classified in Group 4 were reviewed by a consultant radiologist, a consultant urologist, and a urology specialist trainee. Of the 22 patients, 6 (27.2%) had in fact received hormone therapy prior to referral and had their first scan with us for T3 disease.

This left 16 patients with primary bone scans showing possible metastases only outside the pelvis or lumbar spine. On detailed analysis of further imaging and investigation of this group:

- 12 (0.96% of 1201) were false positive bone scans, confirmed by other imaging. One had a focal eccentric rib lesion on X-ray; however, he received local radiotherapy and now is clinically disease-free by ASTRO criteria after four years, with no evidence of metastatic disease in the area described; therefore, this patient was most probably also a false positive (number 12 on Table 2).
- Two (0.17% of 1201) were diagnosed with a second malignancy that explained the findings in the bone scan.
- Two patients (0.17% of 1201, 21-22 in Table 2) had a possible prostatic metastasis outside of the pelvic and lumbar area only, but they had no confirmatory imaging of the suspicious area before death (Table 2).

## 4. Discussion

Little has been published since 1978 on the accuracy of bone scans in the primary diagnosis of CaP [6], yet it remains the most used initial staging investigation. It has been suggested that both MRI [10, 14, 16–18] and CT scan [13] may also be superior to bone scan in assessment of bony metastases. A study by Lecouvet et al. MRI showed a sensitivity of 46% for bone scan alone, 63% for bone scan/targeted X-ray, 83% for bone scan/targeted XR/MRI, and 100% for MRI of the axial skeleton; the corresponding specificities were 32%, 64%, 100%, and 88%, respectively [12]. CT scanning can be useful as a baseline examination in high-risk patients with clinically apparent, grossly advanced local disease, especially to determine lymph node compromise [13]. Also, bone scan gives us no information about the lymph nodes, liver, or direct extraprostatic metastases.

Looking at 1201 initial bone scans from patients newly presenting to our institution, only two patients may have had a single metastasis outside the pelvic/lumbar spine area. None of these patients were in fact felt certain to actually have had a single bone metastasis as seen in the clinical vignettes in Table 1, although in Table 2 where there was uncertainty the patients did have Gleason sum 9 and 10 disease, without any axial imaging. Overall, this suggests that bone scan is unnecessary in a previously untreated man who does not have any bony metastases in the pelvis or lumbar spine [14] on axial imaging.

By contrast, 6 patients previously treated elsewhere who presented to us with hormonally relapsed disease did have eccentric distant metastases, emphasising the unpredictable nature of HRPC.

MR imaging including T1 weighted images of the whole abdomen and pelvis for local and lymph node evaluation maybe sufficient for exclusion of bone and nodal metastases and obviates the need for an additional bone scan. The choice of MRI or CT scan will be dictated by local protocol, but probably according to whether intra- and peri-prostatic information is needed (MRI preferred) or not (CT preferred) [13]. MRI has the ability to detect cellular infiltration of normal marrow and allows early detection of bone metastases before an osteoblastic reaction becomes visible on bone scan and/or targeted X-ray [14]. It seems likely that bone scan will continue to have a role in patients with known metastases, symptomatic bone pain, or hormone relapsed disease

A limitation of our study is that we are unable to reassess the two patients with possible single distant metastases who died without further imaging. Another is that we have relatively small numbers of patients in Groups two and three who had axial imaging, so we are unable to compare the accuracy of these tests to bone scan.

We are proceeding to a regionalized prospective study comparing axial imaging with bone scan in the near future, and hope to be able to confirm these initial results in a multicentre prospective study within the next few years.

## 5. Conclusion

In newly diagnosed prostate cancer, the risk of bone metastases anywhere in the body is negligible if no metastases are seen in the bony pelvis or lumbar spine. Axial imaging (MRI or CT) of the abdomen may be a better single imaging test for staging men with newly diagnosed prostate cancer, with bone scan reserved to assess total metastatic burden in metastatic patients.

## Figures and Tables

**Table 1:** Distribution amongst groups.

Group	Bone scan result	Total	%
Group 1	Negative, no metastases demonstrated	820	68.28%
Group 2	Positive, metastases only in pelvis and/or lumbar spine	136	11.32%
Group 3	Positive, widespread metastasis including pelvis and lumbar spine	223	18.57%
Group 4	Positive, distant metastases without pelvic or lumbar spine abnormalities	22	1.83%

**Table 2:** Analysis of patients classified in Group 4.

	Age	PSA	Alkaline phosphatase	Gleason	Comments
(1)	75	7.3	68	3 + 4	Uptake in 1st left rib anteriorly, negative thorax X-ray, CT scan negative with 5 years of followup.
(2)	79	7.2	54	4 + 3	Uptake in 5th left rib anteriorly, negative CT scan of the chest.
(3)	78	29.7	189	3 + 3	Uptake noted in right shoulder joint/proximal humerus (suspicious), negative shoulder X-ray.
(4)	67	5.0	70	3 + 4	Focus of activity at the right posterior parietal region, negative skull X-ray.
(5)	72	9.7	107	3 + 4	Increased activity at the right superior aspect of the right mandible/right base of the skull. CT scan, X-ray, and MRI compatible with mastoiditis.
(6)	53	19.1	105	3 + 4	Uptake in 8th left rib posteriorly, negative thorax X-ray, stable on two-year followup.
(7)	72	23.5	73	3 + 4	Uptake in 6th left rib posteriorly, negative thorax CT scan, negative MRI.
(8)	78	43.6	87		Focally increased uptake at the lateral third of the left clavicle. Old fractures on X-ray.
(9)	79	15	406	4 + 5	Intense tracer uptake in T11 left side, and 12th rib. Normal CT scan of abdomen-pelvis, confirms Staghorn calculus.
(10)	85	601.2	129	4 + 5	Increased activity at the medial aspect of the right humerus head. No immediate images, negative shoulder X-ray four years later.
(11)	70	56.2	103	3 + 2	Diffuse rib uptake, normal CT and MRI.
(12)	78	11.5	45	4 + 3	Uptake in 8th left rib posteriorly, on review X-ray focal eccentric rib lesion. However treated by radical prostate radiotherapy with clinical response after 4 months (PSA nadir 0.1 ng/mL, 1.1 ng/mL after 4-year followup).

(13)	75	66	62	4 + 3 [8]	Increased uptake in the right 10th rib posteriorly, and left 7th and 10th laterally. Negative CT scan. Previous hormone therapy.
(14)	64	30	94	4 + 5	Intense increased tracer uptake at the right shoulder in the region of the acromium. Previous hormone therapy.
(15)	65	161	137	2 + 2	Multiple regions of inward uptake in left upper scapula left 4th, 6th, 7th, and right 8th ribs anteriorly T6, T9, and T11. Hormone escaped cancer.
(16)	65	9.0	70	3 + 4	Bone scan negative, lung biopsy showed metastatic deposit of prostate cancer. Previously treated with hormones and radiotherapy.
(17)	71	18.4	53	4 + 3 [8]	Focal uptake in the right clavicle and the right posterior 6 rib laterally. Previously treated with hormones and radiotherapy.
(18)	80	57.9	86	3 + 4	Increased uptake at the manubrium suggestive of bone secondaries. Previously treated with hormones and radiotherapy.

(19)	67	7.1	68	4 + 3	Increased in uptake at the right clavicle. Diagnosed with myeloma on bone biopsy.
(20)	84		977	2 + 3	Lesions seen in skull, and thoracic vertebrae. On review compatible with Paget's disease.

**(21)**	**68**	**19.2**	**131**	5 + 5	Increased activity is present at the right tenth rib laterally. No other imaging carried out
**(22)**	**85**	**22.8**	**45**	4 + 5	Increased activity at right 8th rib posteriorly. No other images carried out
